# Isolation of *Vibrio cholerae* and *Vibrio vulnificus* from Estuarine Waters, and Genotyping of *V. vulnificus* Isolates Using Loop-Mediated Isothermal Amplification

**DOI:** 10.3390/microorganisms12050877

**Published:** 2024-04-27

**Authors:** Shin-ichi Miyoshi, Megumi Kurata, Riho Hirose, Masaya Yoshikawa, Yong Liang, Yosuke Yamagishi, Tamaki Mizuno

**Affiliations:** Graduate School of Medicine, Dentistry and Pharmaceutical Sciences, Okayama University, 1-1-1, Tsushima-Naka, Kita-Ku, Okayama-City 700-8530, Okayama, Japan

**Keywords:** *Vibrio cholerae*, *Vibrio vulnificus*, genotype, LAMP, water temperature

## Abstract

Bacteria in the genus *Vibrio* are ubiquitous in estuarine and coastal waters. Some species (including *Vibrio cholerae* and *Vibrio vulnificus)* are known human pathogens causing ailments like cholera, diarrhea, or septicemia. Notably, *V. vulnificus* can also cause a severe systemic infection (known as vibriosis) in eels raised in aquaculture facilities. Water samples were periodically collected from the estuary of the Asahi River, located in the southern part of Okayama City, Japan. These samples were directly plated onto CHROMagar Vibrio plates, and colonies displaying turquoise-blue coloration were selected. Thereafter, polymerase chain reaction was used to identify *V. cholerae* and *V. vulnificus*. A total of 30 *V. cholerae* strains and 194 *V. vulnificus* strains were isolated during the warm season when the water temperature (WT) was higher than 20 °C. Concurrently, an increase in coliforms was observed during this period. Notably, *V. vulnificus* has two genotypes, designated as genotype 1 and genotype 2. Genotype 1 is pathogenic to humans, while genotype 2 is pathogenic to both humans and eels. The loop-mediated isothermal amplification method was developed to rapidly determine genotypes at a low cost. Of the 194 strains isolated, 80 (41.2%) were identified as genotype 1 strains. Among the 41 strains isolated when the WTs were higher than 28 °C, 25 strains (61.0%) belonged to genotype 1. In contrast, of the 32 strains isolated when the WTs were lower than 24 °C, 27 strains (84.4%) belonged to genotype 2. These results suggest that the distribution of the two genotypes was influenced by WT.

## 1. Introduction

Bacteria in the genus *Vibrio* (vibrios) are facultative anaerobic rods that are ubiquitous in seawater and brackish water. Because the abundance of vibrios depends on water temperature (WT), in temperate regions such as Japan, bacteria proliferate vigorously when the WTs are higher than 15–20 °C [1,2]. Therefore, during the warm season, various aquatic organisms including zooplankton, fish, and shellfish are frequently contaminated by vibrios [3,4]. Consumption of contaminated raw or undercooked seafood or shellfish can lead to infectious diseases [5,6]. The genus *Vibrio* comprises approximately 150 species, and at least 12 species have been reported to cause intestinal or extraintestinal infectious diseases in humans [5,6]. Regarding *Vibrio cholerae*, serotypes O1 and O139 are well known for their ability to produce cholerae toxin, which can lead to cholera epidemics (a severe diarrheal disease). Other serotypes may occasionally cause sporadic diarrhea or food poisoning. On the other hand, *Vibrio vulnificus* is known to cause extraintestinal diseases such as primary septicemia or wound infection. Septicemia caused by *V. vulnificus* is opportunistic and often affects patients with underlying conditions, including liver dysfunction, alcoholic cirrhosis, or hemochromatosis [7,8]. In addition, certain strains of *V. vulnificus* cause vibriosis (a lethal hemorrhagic systemic disease) in eels raised in aquaculture facilities [9,10].

*V. vulnificus* has been documented to be classified into two genotypes/genogroups, denoted as genotypes 1 and 2 in the present study. *vvhA* [11], 16S rDNA [12], and *vcg* (virulence-correlated gene) [13,14] have been used as target genes for polymerase chain reaction (PCR)-based genotyping (Table S1). For example, Senoh et al. [11] carried out genotyping targeting *vvhA,* which encodes a hemolytic toxin known as *V. vulnificus* hemolysin (VVH). They observed that most human clinical isolates belonged to genotype 1, characterized by type 1 *vvhA*, whereas all isolates from diseased eels and some human clinical isolates were classified as genotype 2, characterized by type 2 *vvhA*. Notably, genotype 2 also contains environmental isolates that are not pathogenic to experimental eels [11]. Specifically, it has been reported that only strains with large plasmids are pathogenic to experimental eels [15]. These findings suggest that pathogenicity varies between the two genotypes. Therefore, the genotyping appears to be effective for the preliminary assessment of the pathogenicity of *V. vulnificus* isolates. Experiments to clarify the presence or absence of a large plasmid are also needed to evaluate the pathogenicity of genotype 2. In addition to genotyping, biotyping has been used to categorize *V. vulnificus* strains [16]. Senoh et al. [11] further demonstrated that both biotypes 1 and 3 belonged to genotype 1, whereas biotype 2 corresponded to genotype 2.

Loop-mediated isothermal amplification (LAMP) is a rapid, low-cost, and highly specific method for the amplification of target genes. The LAMP assay requires no expensive instruments, and the amplification reactions proceed at a constant temperature. Furthermore, the production of a white precipitate of magnesium pyrophosphate (Mg_2_O_7_P_2_) as a by-product of the reaction enables visible monitoring of the progress of the reaction through increased turbidity. Therefore, LAMP is an effective method for identifying bacterial species, subspecies, and genotypes [17,18].

In this study, estuarine water samples were periodically collected and directly inoculated onto selective plates. Subsequently, putative colonies of *V. cholerae* or *V. vulnificus* were harvested, and the bacterial species were identified using PCR-targeting species-specific genes. In addition, to determine the genotype of *V. vulnificus*, the LAMP method was developed and applied to the isolated strains.

## 2. Materials and Methods

### 2.1. Collection of Water Samples and Measurement of Water Temperatures

We collected environmental water samples from the estuary of the Asahi River (34°35′50.6″ N, 133°57′24.9″ E), located in the southern part of Okayama City, Japan, using a Heyroth water sampler (Sibata Scientific Technology, Saitama, Japan) from April 2013 to August 2018. The water temperature (WT) of the collected samples was immediately measured using a Sanitary Thermo TP-100 MR (Thermoport, Saitama, Japan).

### 2.2. Measurement of Water Quality Parameters

In addition to WT, we measured five physicochemical parameters (salinity, pH, dissolved oxygen (DO), chemical oxygen demand (COD), and ammoniacal nitrogen (NH_4_^+^-N)) and two microbial parameters (standard plate count (SPC) and coliforms). Salinity and pH were assayed using an Eutech Salt 6+ meter (Thermo Fisher Scientific, Waltham, MA, USA) and pH meter F-51 (Horiba, Kyoto, Japan), respectively. DO, COD, and NH_4_^+^-N were measured using Digital Pack Test kits (Kyoritsu Chemical-Check, Tokyo, Japan), whereas SPC and coliforms were enumerated using Microbiological Test Paper sheets (Sibata Scientific Technology).

The correlation between each parameter and the number of vibrios was assessed using regression analysis. We plotted a regression line and calculated the coefficient of determination (R^2^) for each parameter.

### 2.3. Isolation of V. cholerae and V. vulnificus from Water Samples

An aliquot (0.1 mL) of each water sample was inoculated onto CHROMagar Vibrio plates (CHROMagar, Paris, France) supplemented with 0.1% sodium pyruvate (Fujifilm Wako Pure Chemical, Osaka, Japan) and 0.1% soluble starch (Fujifilm Wako Pure Chemical), which may accelerate the resuscitation of viable but nonculturable (VBNC) cells or injured cells in the sample [19,20,21]. Following overnight incubation at 37 °C, the total number of colonies formed was enumerated to estimate the number of vibrios. Thereafter, turquoise-blue colonies on the plate were selected, stamped onto TCBS plates (Eiken Chemical, Tokyo, Japan), and incubated overnight at 37 °C. *V. cholerae* formed yellow colonies on TCBS plates, whereas *V. vulnificus* formed blue-green colonies.

To identify *V. cholerae*, each of the yellow colonies on the plates was suspended in 1 mL of TE buffer (10 mM Tris-HCl, 1 mM EDTA, pH 8.0). The cell suspension was then boiled for 5 min, and the supernatant containing DNA was collected by centrifugation (12,000× *g*, 3 min, 20 °C). The prepared DNA samples were then subjected to PCR targeting *ompW* [22]. Namely, 1 µL of the DNA sample was mixed with 12.5 µL of GoTaq^®^ Green Master Mix (Promega, Madison, WI, USA), 1 µL of 10 µM ompW-F primer (C^64^ACCAAGAAGGTGACTTTATTGTG^97^), 1 µL of 10 µM ompW-R primer (G^633^AACTTATAACCACCCGCG^651^), and 9.5 µL of nuclease-free water. The reaction mixture (25 µL) was heated at 94 °C for 5 min using a Program Temp Control System PC-707 (Astec, Fukuoka, Japan), followed by 30 cycles of PCR (denaturation at 94 °C for 30 s, annealing at 62 °C for 30 s, and extension at 72 °C for 30 s per cycle). After PCR, the products were electrophoresed on a 1.5% agarose gel in TAE buffer (40 mM Tris-acetate, 2 mM EDTA, pH 8.5) at 100 V, and the amplicons were visualized by staining with ethidium bromide (5 µg/mL). The molecular size of the PCR amplicon (588 bp) was determined using the 1 kb Plus DNA Ladder (100 bp to 15 kb) (Thermo Fisher Scientific).

PCR targeting *ctxA* [23] was also performed to determine the presence or absence of the *ctxAB* gene. Briefly, 1 µL of the DNA sample, 12.5 µL of GoTaq^®^ Green Master Mix (Promega), 1 µL of 10 μM ctxA-1 primer (C^197^TCAGACGGGATTTGTTAGGCAC^219^), 1 µL of 10 µM ctxA-2 primer (T^474^CTATCTC TGTAGCCCCTATTACG^498^), and 9.5 µL of nuclease-free water were mixed and heated at 94 °C for 5 min. Thereafter, the reaction mixture was subjected to 30 cycles of PCR (denaturation at 94 °C for 30 s, annealing at 64 °C for 30 s, and extension at 72 °C for 30 s per cycle). The PCR products were electrophoresed on a 1.5% agarose gel in TAE buffer at 100 V, and the amplicons were visualized by staining with ethidium bromide.

For identification of *V. vulnificus*, each of the blue-green colonies on TCBS plates was suspended in 1 mL of TE buffer. The cell suspension was then boiled for 5 min, and the supernatant containing DNA was collected by centrifugation (12,000× *g*, 3 min, 20 °C). Then, the prepared DNA samples were subjected to PCR targeting *vvhA* [24]. Namely, 1 µL of the DNA sample was mixed with 12.5 µL of GoTaq^®^ Green Master Mix (Promega), 1 µL of 10 μM vvcont-F primer (C^41^CGCGGTACAGGTTGGCGCA^60^), 1 µL of 10 µM vvcont-R primer (C^541^GCCACCCACTTTCGGGCC^559^), and 9.5 µL of nuclease-free water. The reaction mixture (25 µL) was heated to 94 °C for 5 min, followed by 30 cycles of PCR (denaturation at 94 °C for 30 s, annealing at 66 °C for 30 s, and extension at 72 °C for 90 s per cycle). After PCR, the products were electrophoresed on a 1.5% agarose gel in TAE buffer at 100 V, and the amplicons were visualized by staining with ethidium bromide.

### 2.4. LAMP for Identification of V. vulnificus Genotypes

Ten strains of *V. vulnificus*, comprising five genotype 1 strains (strain L-180, N-87, KI-50, 374, and KI-5) and five genotype 2 strains (strain CDC B3547, MLT401, E86, CECT5198, and YN-03) [11] (Table 1), were inoculated into 5 mL of Luria-Bertani (LB) broth (1.0% tryptone [Becton, Dickinson and Company, Franklin Lakes, NJ, USA], 0.5% yeast extract [Becton, Dickinson and Company], 2.0% NaCl, pH 7.0), and the bacteria were grown with shaking until OD_600_ reached 0.5.

After cultivation, bacterial cells were harvested by centrifugation at 2300× *g* for 15 min and suspended in 2 mL of PBS (137 mM NaCl, 8.10 mM Na_2_HPO_4_, 2.68 mM KCl, 1.47 mM KH_2_PO_4_, pH 7.4). The cell suspension was centrifuged again at 2300× *g* for 15 min, and bacterial cells were collected. Subsequently, the cells were mixed with 267 µL of TE buffer, 30 µL of 10% SDS, and 3 µL of 20 mg/mL proteinase K (Sigma-Aldrich, St. Louis, MO, USA), followed by incubation at 37 °C for 1 h to lyse bacterial cells. The cell lysate was mixed with 50 µL of 5 M NaCl and 40 µL of 10% cetyltrimethylammonium bromide (CTAB)-4% NaCl and incubated at 65 °C for 10 min. Following the addition of 390 µL of chloroform-isoamyl alcohol (24:1), the mixture was centrifuged at 27,000× *g* for 5 min. The supernatant containing DNA was collected, mixed with 3 µL of RNase A (Sigma-Aldrich), and incubated at 37 °C for 1 h to digest any contaminating RNA. Thereafter, DNA was precipitated by adding ethanol, collected by centrifugation, and dissolved in TE buffer at concentrations ranging from 12.5 to 100 ng/µL. The prepared DNA samples were used for the LAMP assay.

**Figure 1:**
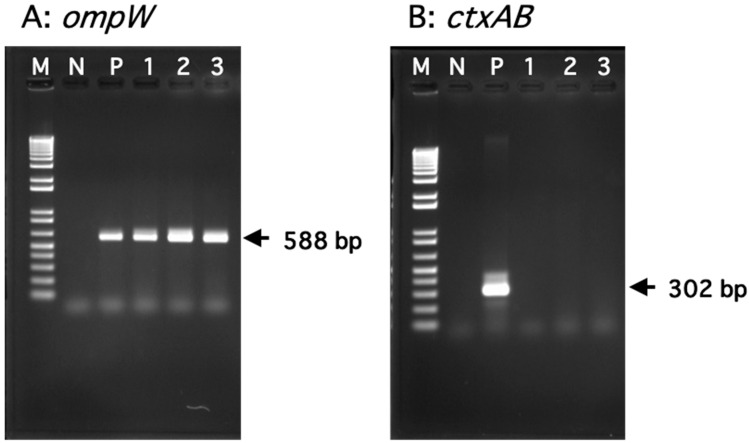
Representative results of agarose gel electrophoresis for the PCR products to detect *V. cholerae* genes. (**A**) Detection of the *ompW* gene. Lane M: 1 Kb Plus DNA Ladder (100 bp to 15 kb); lane N: *V. mimicus* strain CS-66 (negative control); lane P: *V. cholerae* strain NCTC4715 (positive control); lanes 1–3: *V. cholerae* strains isolated from estuarine waters. (**B**) Detection of the *ctxAB* genes. Lane M: 1 kb Plus DNA Ladder (100 bp to 15 kb); lane N: *V. cholerae* strain NCTC4715 (negative control); lane P: *V. cholerae* strain N16961 (positive control); lanes 1–3: *V. cholerae* strains isolated from estuarine waters.

For the LAMP assay, the Loopamp^®^ DNA amplification kit and Loopamp reaction tubes were obtained from Eiken Chemical (Tokyo, Japan). Two sets of primers (FIP [F1c + F2], BIP [B1c + B2], F3 primer, and B3 primer) (Table 2) were designed based on specific regions (F3, F2, F1, B1c, B2c, and B3c) in *vvhA* (Figure 1). The reaction mixture (25 µL) was prepared as follows: 2.0 µL of the DNA sample (25 to 200 ng) was mixed with 12.5 µL of 2 x Reaction Mix, 1.0 µL of each primer [40 pmol of FIP, 40 pmol of BIP, 5 pmol of F3 primer, 5 pmol of B3 primer], 1.0 µL of Bst DNA Polymerase (8 U), and 5.5 µL of nuclease-free water. The amplification reaction was performed at 63 °C for 1 h and monitored using a real-time turbidity meter (LoopampEXIA, Eiken Chemical). The increase in turbidity at 650 nm (A_650_) resulting from the production of insoluble Mg_2_O_7_P_2_ was measured in real time. The Tt value (min), which represents the reaction time required for the detection of Mg_2_O_7_P_2_, was then determined. Using LoopampEXIA, the LAMP reaction and turbidity measurements could be performed simultaneously. The LAMP amplification reaction was terminated by heating at 80 °C for 5 min. In some experiments, the products were electrophoresed on a 1.5% agarose gel in TAE buffer at 100 V, and the amplicons were visualized by staining with ethidium bromide.

### 2.5. Genotyping of V. vulnificus Strains Isolated from the Estuarine Waters

Each of the isolated *V. vulnificus* strains was cultivated on a CHROMagar Vibrio plate, and a representative colony was selected and suspended in 1 mL of TE buffer. The cell suspension was then boiled for 5 min, and the supernatant containing DNA was collected by centrifugation. Genotyping was performed using the LAMP assay targeting *vvhA*.

## 3. Results and Discussion

### 3.1. Isolation of V. cholerae and V. vulnificus from the Estuarine Waters

Estuarine water samples were directly inoculated on CHROMagar Vibrio plates. After overnight incubation at 37 °C, the number of colonies was counted to estimate the total number of vibrios (Table 3). Vibrios were frequently isolated from water samples collected during the warm season (June to September). The regression analysis between WT and the number of vibrios resulted in an R^2^ of 0.60 for WT. However, it is noteworthy that on several occasions, vibrios were not detected (CFU/mL was less than 10), even during the warm season, suggesting that fluctuations (increase and decrease) in vibrios occur at short intervals in the estuarine waters.

In the present study, we used CHROMagar Vibrio plates supplemented with sodium pyruvate and soluble starch, which are known to accelerate the resuscitation of VBNC or injured cells [19,20,21]. However, no strain was isolated from water samples collected during the cold season (December to March). We recently found that some proteolytic enzymes, including trypsin, drastically affect the recovery of *V. cholerae* from the VBNC state [25]. Therefore, the addition of proteolytic enzymes to the plate may facilitate the isolation of vibrios during the cold season.

From CHROMagar Vibrio plates, turquoise-blue colonies, possibly of *V. cholerae* or *V. vulnificus*, were harvested and stamped on TCBS agar plates. To identify *V. cholerae*, yellow colonies were selected, and PCR was performed targeting *ompW,* which encodes the *V. cholerae*-specific outer membrane protein OmpW [22]. Thirty colonies showed positive PCR results and were thus confirmed to be *V. cholerae* (Table 3). The identified *V. cholerae* strains were also examined for the presence of *ctxAB,* which encodes cholera toxin, but none of them yielded positive results. Representative results of the PCR experiments to detect *V. cholerae ompW* or *ctxAB* are shown in Figure 1.

**Figure 2:**
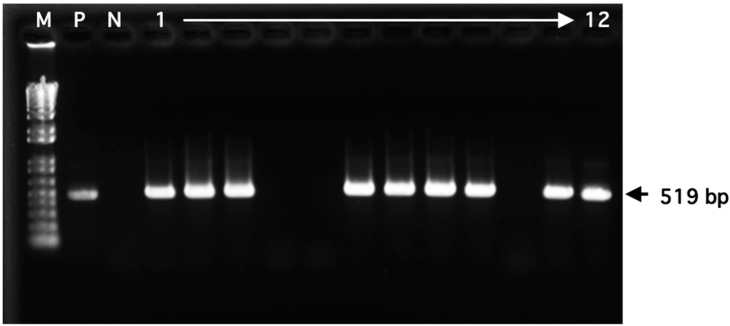
Representative results of agarose gel electrophoresis for the PCR products to detect *V. vulnificus vvhA* gene. Lane M: 1 kb Plus DNA Ladder (100 bp to 15 kb); lane P: *V. vulnificus* strain L-180 (positive control); lane N: *V. cholerae* strain NCTC4715 (negative control); lanes 1–12: Vibrios isolated from estuarine waters.

To identify *V. vulnificus*, the blue-green colonies on TCBS plates were subjected to PCR targeting *vvhA* (Figure 2)*,* which encodes the hemolytic toxin VVH [24]. Although 194 colonies were identified as *V. vulnificus* (Table 4), this bacterium was isolated between May and October. Especially, 83 strains (42.8%) were isolated in July. Additionally, we observed that *V. vulnificus* was isolated only when the WTs were higher than 20 °C, with 87 strains (44.8%) isolated at 26–28 °C (Table 5).

Therefore, in line with previous studies [1,2,26], WT was found to be an important environmental parameter for the isolation of *V. cholerae* and *V. vulnificus*.

### 3.2. Water Quality Parameters of the Water Samples

In addition to the WT, five physicochemical and two microbial parameters were measured (Table 3). Among these parameters, only coliform levels increased during the warm season. This observation suggests a potential overlap in the distribution of vibrios and coliforms in estuarine waters; however, the R^2^ was as low as 0.11. Conversely, DO levels were slightly lower during periods of higher WT, likely because of the decreased solubility of oxygen as the temperature increased. Specifically, the saturated concentration of DO is 10.92 mg/L at 10 °C but decreases to 8.11 mg/L at 25 °C. None of the other parameters showed seasonal changes.

### 3.3. Development of a Genotype-Specific LAMP Method Targeting vvhA

**Figure 3:**
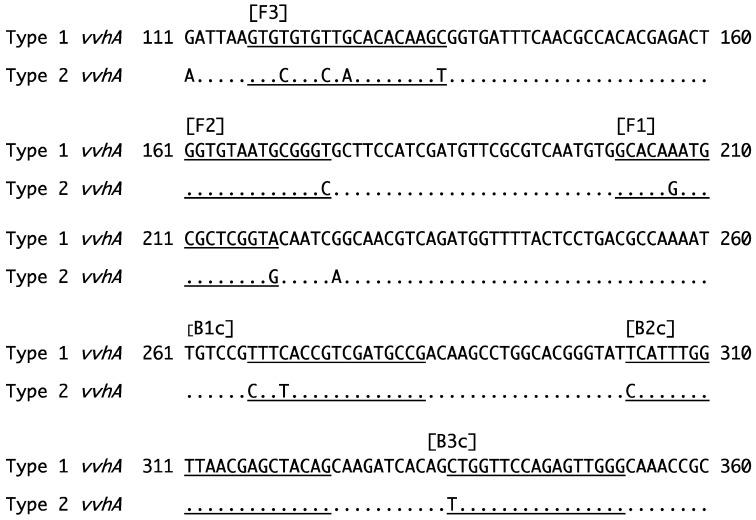
Partial nucleic acid sequences of type 1 *vvhA* of strain L-180 (a genotype 1 strain) and type 2 *vvhA* of strain CDC B3547 (a genotype 2 strain). The underlines indicate the positions of the primers (F3, F2, F1, B1c, B2c, and B3c) for the LAMP assay.

*V. vulnificus* is classified into two genotypes, genotypes 1 and 2, and several genes including *vvhA* [11] have been utilized for PCR-based genotyping. To develop the LAMP-based genotyping system, two sets of primers (Table 2) were designed from type-specific regions within the *vvhA* gene of strain L-180 [11], a genotype 1 strain with type 1 *vvhA* (accession number AB124802), and strain CDC B3547 [11], a genotype 2 strain with type 2 *vvhA* (accession number AB124803) (Figure 3).

**Figure 4:**
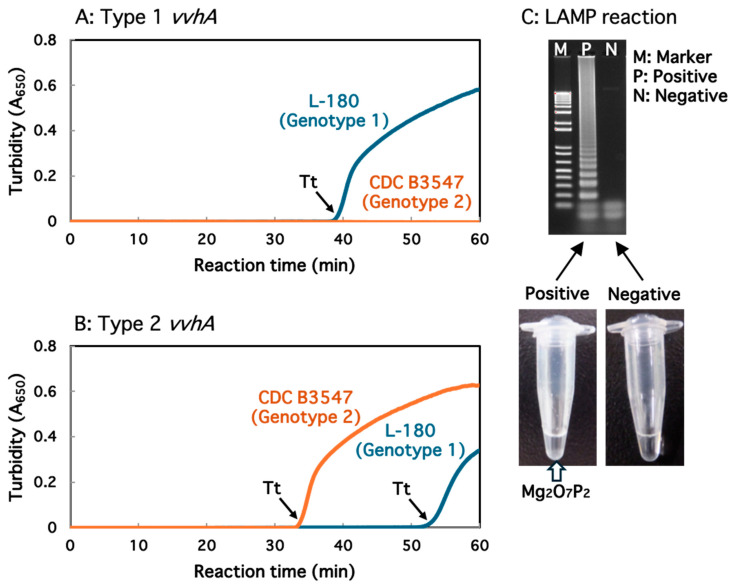
Type-specific amplification of the *vvhA* genes using the LAMP assay. The DNA sample (25 ng) was prepared from strain L-180 or CDC B3547, and the amplification reaction was performed at 63 °C for 1 h. The increase in the A_650_ caused by the production of insoluble Mg_2_O_7_P_2_ was measured in real-time, and the Tt value was determined. (**A**) The LAMP assay to amplify type 1 *vvhA*. (**B**) The LAMP assay to amplify type 2 *vvhA*. (**C**) Production of insoluble Mg_2_O_7_P_2_ in the positive tube, followed by detection of the amplified products using agarose gel electrophoresis.

When an appropriate amount of DNA (25 to 200 ng) prepared from strain L-180 was subjected to the LAMP assay using primers designed for type 1 *vvhA*, gene amplification was observed at approximately 37 min in all DNA samples (Table 6). This indicates that Tt values were minimally affected by the amount of DNA used, suggesting that the amplification reaction approached saturation at 25 ng of DNA. Tian et al. [27] reported that the sensitivity of LAMP targeting *V. vulnificus gyrB* was only 10 fg/µL. Although we did not carry out a sensitivity test, the sensitivity of LAMP targeting *vvhA* may be comparable at a similar concentration. In contrast, in the LAMP assay using the primers for type 2 *vvhA*, amplification of *vvhA* required approximately 50 min (Table 6). In contrast, when DNA from strain CDC B3547 was used, the *vvhA* gene was amplified at 30–32 min using primers designed for type 2 *vvhA* but not for type 1 *vvhA* (Table 6). Figure 4 illustrates representative results of the LAMP assay using 25 ng of the DNA samples prepared from strains L-180 and CDC B3547.

The LAMP assay using the primers for type 1 *vvhA* exhibited consistent gene amplification at approximately 40 min when DNA samples (25 ng) from the genotype 1 strains (strains L-180, N-87, KI-50, 374, and KI-5) were utilized (Table 7). Conversely, in the assay with DNA samples (25 ng) from genotype 2 strains, *vvhA* was not amplified even though the reaction time was longer than 50 min. In contrast, in the LAMP assay using primers for type 2 *vvhA*, only DNA samples from genotype 2 strains (strain CDC B3547, MLT401, E86, CECT5198, and YN-03) showed positive results within 35 min (Table 7).

Furthermore, to examine whether the developed LAMP method is also effective with crude DNA samples, strain L-180 was cultivated in LB broth until the OD_600_ reached 0.5, and the culture was subsequently boiled for 5 min. Thereafter, the supernatant containing DNA was collected by centrifugation and subjected to the LAMP assay. Using primers specific to type 1 *vvhA*, amplification of the gene was observed after approximately 35 min. However, when primers specific to type 2 *vvhA* were used, the amplification of the gene required more than 50 min. Therefore, LAMP-based genotyping was also found to be effective in crude DNA samples.

Additionally, genomic DNA was extracted from other bacterial species, namely *Vibrio mimicus* strain CS-66, *Vibrio parahaemolyticus* strain AQ3436, and *Escherichia coli* strain B, using the CTAB method. Each DNA sample (25 ng) was then subjected to the LAMP assay. However, neither genotyping system yielded a positive result.

Taken together, it may be concluded that the LAMP method targeting *vvhA* is specific to *V. vulnificus* and suitable for genotyping bacterial species. Although several researchers have developed LAMP methods targeting *vvhA* or other genes [27,28,29], none have been applied to differentiate *V. vulnificus* isolates into genotypes.

### 3.4. Genotyping of V. vulnificus Strains Isolated from the Estuarine Waters

Each strain was cultivated on a CHROMagar Vibrio plate, and a representative colony was selected, suspended in TE buffer, and boiled for 5 min to obtain the crude DNA preparation. The results of the LAMP-based genotyping showed that out of 194 environmental strains of *V. vulnificus* isolated in the present study, 80 strains (41.2%) were identified as genotype 1, and 114 strains (58.8%) were classified as genotype 2. The specificity of the LAMP method was verified by PCR targeting *vcg* [13,14] or *vvp/vvpE* encoding a metalloprotease VVP [30]. We found that the LAMP method yielded genotyping results identical to those of the PCR methods.

Seasonal variations of the two genotypes were compared (Table 4). Although a total of 51 strains were isolated in the early warm season (May and June), only nine strains (17.6%) belonged to genotype 1. In contrast, among the 39 strains isolated in August (the middle of the warm season), 23 strains (59.0%) were genotype 1. As shown in Table 5, 41 strains were isolated when the WTs were 28–31 °C, and, among them, 25 strains (61.0%) were genotype 1. A total of 32 strains were isolated when the WTs were lower than 24 °C. Of these strains, 27 strains (84.4%) were genotype 2. These results suggest that the distribution of the two genotypes depends on the WT.

The genotype 1 strains of *V. vulnificus* are pathogenic exclusively to humans, whereas the genotype 2 strains are pathogenic to both eels and humans [9,11]. The present study indicated that genotype 2 predominated in slightly cold (22 °C to 26 °C) estuarine waters. This finding suggests that the optimal growth temperature of genotype 2 was lower than that of genotype 1. Therefore, only genotype 2 may cause vibriosis in eels raised in aquaculture facilities. To clarify this point, it may be necessary to genotype the *V. vulnificus* strains inhabiting such farmed eels.

## 4. Conclusions

Estuarine water samples were periodically collected and directly inoculated onto CHROMagar Vibrio plates. A total of 30 *V. cholerae* strains and 194 *V. vulnificus* strains were isolated during the warm season when WTs exceeded 20 °C. Among the water quality parameters measured, coliform levels also increased during the warm season.

To determine the genotype of the isolated *V. vulnificus* strains, a LAMP method targeting *vvhA* was developed. Among the 194 strains, genotypes 1 and 2 comprised 80 (41.2%) and 114 (58.8%) strains, respectively. Of the 41 strains isolated when the WTs were 28–31 °C, 25 (61.0%) strains were genotype 1. In contrast, of the 32 strains isolated when the WTs were lower than 24 °C, 27 (84.4%) were genotype 2. These results suggest that the distribution of these two genotypes depends on WT.

*V. cholerae* and *V. vulnificus* are important causative agents of food-borne diseases. In the present study, we established methods for the simultaneous detection of these two *Vibrio* species in estuarine waters and for the rapid determination of the genotype of *V. vulnificus*. The developed methods may be useful for assessing the potential hazards associated with seawater, brackish water, or seafood.

## Figures and Tables

**Table 1:** *V. vulnificus* strains used for the * LAMP assay.

Strain	Source	Country	*vvhA*	Reference
Genotype 1				
L-180	Human blood	Japan	Type 1	[11]
N-87	Human blood	Japan	Type 1	[11]
KI-50	Brackish water	Japan	Type 1	[11]
374	Human blood	USA	Type 1	[11]
KI-5	Brackish water	Japan	Type 1	[11]
Genotype 2				
CDC B3547	Human leg ulcer	USA	Type 2	[11]
MLT401	Seawater	USA	Type 2	[11]
E86	Diseased eel	Spain	Type 2	[11]
CECT5198	Diseased eel	Spain	Type 2	[11]
YN-03	Human blood	Japan	Type 2	[11]

* Loop-mediated isothermal amplification.

**Table 2:** Primers for the * LAMP assay targeting to *vvhA*.

Primer	Sequence (5′-3′)	Tm (°C)
Type 1 *vvhA*		
FIP (F1c + F2)	TACCGAGCGCATTTGTGCGACTGGTGTAATGCGGGT	85.9
F3 primer	GTGTGTGTTGCACACAAGC	61.6
BIP (B1c + B2)	TTTCACCGTCGATGCCGCTGTAGCTCGTTAACCAAATGA	84.1
B3 primer	CCCAACTCTGGAACCAG	59.1
Type 2 *vvhA*		
FIP (F1c + F2)	CACCGAGCGCATCTGTGACTGGTGTAATGCGGGC	86.6
F3 primer	GTGCGTGCTACACACAAGT	60.0
BIP (B1c + B2)	CTTTACCGTCGATGCCGACTGTAGCTCGTTAACCAAATGG	81.7
B3 primer	CCCAACTCTGGAACCAA	59.6

* Loop-mediated isothermal amplification.

**Table 3:** Values of water quality parameters and numbers of vibrios in the water samples.

Month	WT (°C)	pH	Salinity (%)	DO (mg/L)	COD (mg/L)
January	4.7–8.3	7.7–8.4	0.7–2.0	11.0	5–12
February	5.7–8.6	7.9–8.1	1.2–1.4	7.8–10.6	5–12
March	5.3–6.7	8.0–8.1	0.9–1.4	9.8–11.0	5–7
April	11.8–16.2	7.3–8.4	0.5−2.6	9.4−11.0	6−11
May	15.4−23.3	6.9−8.5	0.8−1.5	7.6−9.0	6−10
June	23.7−26.1	7.2−8.7	0.7−1.3	5.4−10.6	6−10
July	25.1−28.8	7.0−8.6	0.4−1.7	7.2−11.0	6−11
August	26.4−30.2	6.8−8.5	0.4−1.6	7.4−8.8	5−11
September	23.0−26.5	7.4−8.6	0.6−1.6	8.6−10.8	6−10
October	16.5−24.6	7.2−8.8	0.3−1.2	7.8−10.8	5−9
November	10.1−17.5	7.5−8.2	0.9−2.0	8.2−10.8	4−8
December	6.0−12.8	7.2−8.4	0.8−1.6	9.4−11.0	5−17
**Month**	**NH_4_^+^-N (mg/L)**	**Coliforms (CFU/mL)**	**SPC (CFU/mL)**	**Vibrios (CFU/mL)**	**Number of *V. cholerae***
January	0.1−0.2	<1−70	60−340	<10	0
February	0.1−0.2	<1−20	100−220	<10	0
March	0.0−0.2	<1−50	170−320	<10	0
April	0.1−0.5	<1−40	70−1950	<10−20	0
May	0.1−0.5	<1−680	60−560	<10−50	6
June	0.1−0.5	10−2210	300−720	<10−260	10
July	0.1−0.2	10−2330	190−1290	<10−460	4
August	0.2	<1−210	120−800	10−200	0
September	0.1−0.2	10−200	80−580	<10−180	6
October	0.1−0.5	<1−800	110−790	<10−40	4
November	0.1−0.2	<1−140	140−1000	<10−110	0
December	0.0−0.2	<1−420	220−390	<10	0

**Table 4:** Numbers of *V. vulnificus* isolated from estuarine waters.

Genotype 1	Genotype 2	Total
0	4	4
9	38	47
38	45	83
23	16	39
7	10	17
3	1	4
80	114	194

**Table 5:** Water temperature and isolation of *V. vulnificus*.

WT (°C)	Genotype 1	Genotype 2	Total
20–22	1	0	1
22–24	4	27	31
24–26	12	22	34
26–28	38	49	87
28–31	25	16	41
Total	80	114	194

**Table 6:** Type-specific amplification of *vvhA* using LAMP.

DNA (ng)	Tt value (min)	
	Type 1 *vvhA*	Type 2 *vvhA*
Strain L-180 (Genotype 1)		
0	>50.0	>50.0
25	37.4	49.9
50	36.6	>50.0
100	36.8	49.6
200	36.6	>50.0
Strain CDC B3547 (Genotype 2)		
0	>50.0	>50.0
25	>50.0	32.1
50	>50.0	31.7
100	47.1	30.9
200	44.9	31.0

**Table 7:** Genotype specificity of LAMP targeting *vvhA*.

Strain	* Tt Value (min)	
	Type 1 *vvhA*	Type 2 *vvhA*
Genotype 1		
L-180	40.1	>50.0
N-87	40.9	>50.0
KI-50	37.4	>50.0
374	39.0	> 50.0
KI-5	38.6	>50.0
Genotype 2		
CDC B3547	>50.0	34.6
MLT401	>50.0	35.3
E-86	>50.0	33.6
CECT5198	>50.0	34.6
YN-03	>50.0	34.7

* The reaction time required for Mg_2_O_7_P_2_ production.

## Data Availability

Data are contained within the article and Supplementary Materials.

## Supplementary Materials

The following supporting information can be downloaded at: https://www.mdpi.com/article/10.3390/microorganisms12050877/s1, Table S1. Types of genes in genotypes 1 and 2 of *V. vulnificus*.
