# Supplementary material for: Isolation of Vibrio cholerae and Vibrio vulnificus from Estuarine Waters, and Genotyping of V. vulnificus Isolates Using Loop-Mediated Isothermal Amplification
Source: Microorganisms. 2024 Apr 27;12(5):877. doi: 10.3390/microorganisms12050877 (PMC11124270; doi:10.3390/microorganisms12050877)
Supplement: Supplementary file 1 [file microorganisms-12-00877-s001.zip › microorganisms-2957963-supplementary.pdf]

Supplementary Materials

Table S1. Types of genes in genotypes 1 and 2 of *V. vulnificus*

*Genotype	Biotype	Type of the gene		
		vvhA **gene**	**16S rDNA**	***vcg*** **gene**
1	1 and 3	Type 1	Type B	Type C
2	2	Type 2	Type A	Type E

* Strains in genotype 1 are pathogenic to human, whereas some strains in genotype 2 (having a large plasmid) are pathogenic to eels and human.
